# Reevaluating the ability of cerebellum in associative motor learning

**DOI:** 10.1038/s41598-019-42413-5

**Published:** 2019-04-15

**Authors:** Da-bing Li, Juan Yao, Lin Sun, Bing Wu, Xuan Li, Shu-lei Liu, Jing-ming Hou, Hong-liang Liu, Jian-feng Sui, Guang-yan Wu

**Affiliations:** 1 Experimental Center of Basic Medicine, College of Basic Medical Sciences, Army Medical University, Chongqing, 400038 China; 2 Department of Physiology, School of Basic Medical Sciences, Southwest Medical University, Luzhou Sichuan, 646000 China; 3 Department of Physiology, College of Basic Medical Sciences, Army Medical University, Chongqing, 400038 China; 4 Department of Rehabilitation, Southwest Hospital, Army Medical University, Chongqing, 646000 China

## Abstract

It has been well established that the cerebellum and its associated circuitry constitute the essential neuronal system for both delay and trace classical eyeblink conditioning (DEC and TEC). However, whether the cerebellum is sufficient to independently modulate the DEC, and TEC with a shorter trace interval remained controversial. Here, we used direct optogenetic stimulation of mossy fibers in the middle cerebellar peduncle (MCP) as a conditioned stimulus (CS) replacement for the peripheral CS (eg, a tone CS or a light CS) paired with a periorbital shock unconditioned stimulus (US) to examine the ability of the cerebellum to learn the DEC and the TEC with various trace intervals. Moreover, neural inputs to the pontine nucleus (PN) were pharmacological blocked to limit the associative motor learning inside the cerebellum. We show that all rats quickly acquired the DEC, indicating that direct optogenetic stimulation of mossy fibers in the left MCP is a very effective and sufficient CS to establish DEC and to limit the motor learning process inside the cerebellum. However, only five out of seven rats acquired the TEC with a 150-ms trace interval, three out of nine rats acquired the TEC with a 350-ms trace interval, and none of the rats acquired the TEC with a 500-ms trace interval. Moreover, pharmacological blocking glutamatergic and GABAergic inputs to the PN from the extra-cerebellar and cerebellar regions has no significant effect on the DEC and TEC learning with the optogenetic CS. These results indicate that the cerebellum has the ability to independently support both the simple DEC, and the TEC with a trace interval of 150 or 350 ms, but not the TEC with a trace interval of 500 ms. The present results are of great importance in our understanding of the mechanisms and ability of the cerebellum in associative motor learning and memory.

## Introduction

A prerequisite to understand the neural mechanisms by which an organism acquires and retains information is the identification of the neural substrates of the learning and memory^1^. It now appears that different forms or aspects of learning and memory rely on distinct but distributed neural substrates and circuits^2,3^. For example, the hippocampus appears to be important for spatial, contextual and relational memories^4–6^, whereas the amygdala is a key brain structure involved in the acquisition and expression of fear conditioning^7–10^. By contrast, the cerebellum plays a pivotal role in an associative motor learning^11–14^. For biological analysis, eyeblink conditioning (EBC), a form of associative motor learning paradigm, provides an important advantage over complex forms of learning and memory in that the stimuli involved are well defined and can be precisely modulated and has proven particularly useful for studying the neural mechanisms underlying associative motor learning^1,15–19^.

Converging lines of evidence from lesion, reversible inactivation, genetic manipulation, electrical stimulation, optogenetic inhibition or activation, electrophysiological recording, and brain-imaging studies indicate that the cerebellum is essential for acquisition, expression, and extinction of the EBC^13,20–31^. McCormick *et al*. first showed that electrolytic lesions of the ipsilateral cerebellum completely prevented the acquisition and retrieval of the delay eyeblink conditioning (DEC), where the conditioned stimulus (CS) is presented before the unconditioned stimulus (US) and the two stimuli end together^32,33^. Their later study also showed that smaller electrolytic lesions of the ipsilateral dentate-interpositus nuclei abolished the retrieval of DEC^34^. In addition, inactivation of the anterior interpositus nucleus by lidocaine, cooling, or muscimol reversibly abolished the DEC^35–38^. Multiple-unit and extracellular single unit recordings further demonstrated that neurons in the interpositus nucleus not only showed a wide diversity in latencies and patterns of response to single corneal air puff stimulation in the alert cat^39^ but also exhibited a burst of firing in learned animals during eyeblink conditioning^40–43^. Although cerebellar cortex lesions studies showed inconsistent results^44–49^, studies of cerebellar Purkinje cells have revealed inhibitory or excitatory patterns of cellular activity that related to CS or US presentation, conditioned response (CR) generation, and the timing of the CR^14,50–56^. These data, together with data from a number of other studies, has provided clear evidence that the cerebellum is necessary for the simple DEC. However, whether the cerebellum is sufficient to mediate simple DEC still remain controversy.

One of the bits of evidence to demonstrate cerebellum’s sufficient ability for DEC learning comes from a series studies using decerebrate animals to establish the simple DEC^57–62^. It was found that decerebrate animals could readily acquire the DEC that were similar to those reported previously in various intact animals, suggesting that the cerebellum and brainstem may be essential and sufficient to mediate the simple DEC. However, these decerebrate animals might still have small remnant of the forebrain or midbrain. Another piece of evidence comes from a number of studies using electrical stimulation of the cerebellum or its input fibers as the CS or US to limit DEC learning inside cerebellum^25,63–66^. However, the electrical stimulation CS might be not “pure” because of the possibility of electrical current diffusion into extra-cerebellar regions in these studies. Moreover, these studies did not block the possible inputs form extra-cerebellar regions to the PN. Together, to confirm that cerebellum comprises essential and sufficient site for the simple DEC, it is necessary but challenging to exclude the influence of the extra-cerebellar regions. In addition, it has also been proposed that the posterior interpositus nucleus is mainly related to the proper performance of eyeblink learned responses but not to the acquisition process of eyeblink responses^67–72^. On the other hand, lidocaine inactivation of the motor cortex evoked a significant decrease in learning curves and in the amplitude of CRs, while the electrical train stimulation of the motor cortex can generate CRs similar to those that are naturally evoked in rabbits, suggesting the motor cortex are involved in the acquisition and expression of the DEC^73^.

Previous studies indicated that the brainstem-cerebellar circuit is essential and perhaps sufficient to modulate the DEC under optimal learning conditions, whereas several forebrain structures, including the hippocampus and the medial prefrontal cortex (mPFC), are required additionally to modulate trace eyeblink conditioning (TEC), in which there is a stimulus-free trace interval between the CS and the US^74–83^. Lesion studies showed that hippocampus is engaged in TEC when the trace interval is 250 ms or more in rodents, and at least 500 ms in rabbits^81–83^, suggesting that the cerebellum may be able to independently support the TEC with a shorter trace interval. Moreover, our recent study indicates that muscimol inactivation of the anterior cingulate cortex one day after learning had no significant effects on retrieval of the TEC with 350-ms trace interval in guinea pigs when louder CS (100-dB) was used^84^, indicating that the cerebellum is capable of supporting the TEC with a relative shorter trace interval when sufficient CS and US signals into the cerebellum are provided. However, in these lesion and inactivation studies, other extra-cerebellar regions, excepting hippocampus and mPFC, remained intact and might be involved in modulation of the TEC. Thus, the ability of cerebellum to independently support the TEC could not be inferred from these indirect evidences. To elucidate this question, it is necessary to limit EBC learning process merely inside the cerebellum. Kalmbach *et al*. used electrical stimulation of mossy fibers in the middle cerebellar peduncle as a CS in place of the peripheral CS and demonstrated that rabbits could learn the TEC with a trace interval of 200, 300, or 400 ms, but not of 500 ms^25^, supporting the assumption of that the cerebellum could independently support EBC learning when the activities of the mossy fiber and climbing fiber approximately overlap in time (or nearly so)^25,85,86^. However, in this study, electrical stimulation of mossy fibers as a CS might be not “pure” due to any possible spread of electrical current. For example, the spread of electrical current may also stimulate the meninges, leading to a somatosensory CS, which has been proven to be an effective and sufficient CS for EBC^12,87^. Thus, the maximal trace interval for TEC to be established without involvement of extra-cerebellar regions remains unknown.

In the present study, in order to preclude contributions to DEC and TEC from the extra-cerebellar regions, we used direct optogenetic stimulation of mossy fibers in the MCP as a CS replacement for the peripheral CS (eg, a tone or a light CS) paired with a periorbital shock US and reversibly block external glutamatergic and GABAergic inputs to the pontine nucleus (PN). The results revealed that the cerebellum can independently support learning of the simple DEC and TEC with a trace interval of 150 and 350 ms.

## Results

### The optogenetic CS was sufficient for the acquisition of the simple DEC in all rats

It is generally accepted that the cerebellum comprises essential and sufficient sites for supporting the DEC under optimal learning conditions^21,88,89^, whereas some studies also reported that components of the auditory and visual CS pathways, including the medial auditory thalamic nuclei, inferior colliculus, lateral geniculate nucleus, superior colliculus, play an important role in the simple DEC^11,90–93^. Moreover. in the EBC, the CS signals are conveyed to the cerebellum via mossy fiber inputs, and electrical stimulation of mossy fibers has been shown to substitute for a peripheral CS to support EBC in rabbits^25,64,66,94–96^. Here, we first examine whether the optogenetic CS is sufficient for the acquisition of the simple DEC.

**Figure 1:**
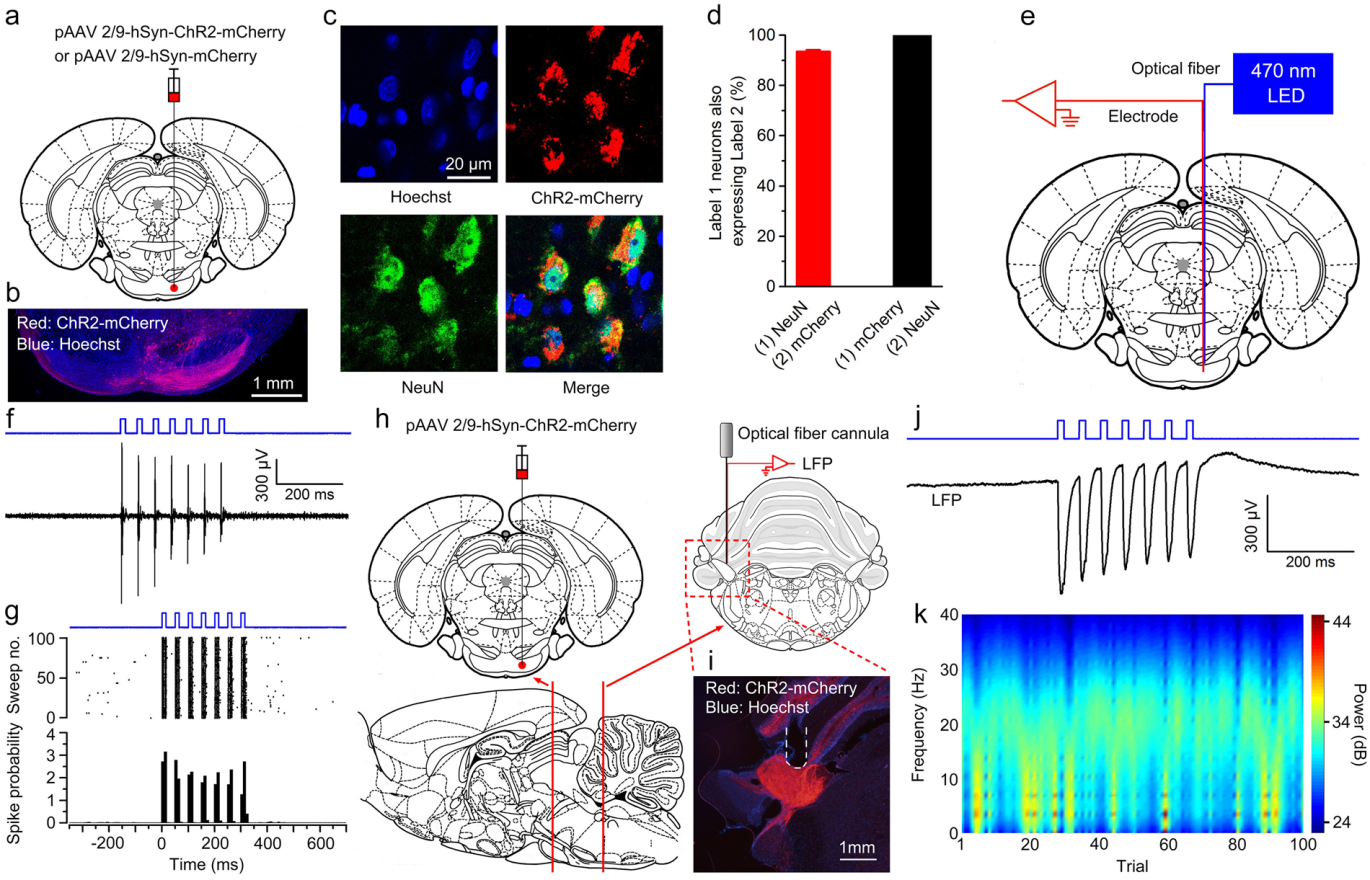
Selective labelling the right PN neurons and optogenetic stimulation of mossy fibers in the left MCP. (**a**) The rats were stereotactically injected with pAAV 2/9-hSyn-ChR2-mCherry or pAAV 2/9-hSyn-mCherry targeting the right PN. (**b**) Example of ChR2-mCherry expression in the right PN. (**c**) Representative images showing cell-specific ChR2-mCherry expression (red) in neurons (green) of the PN. (**d**) Statistics of expression in the neurons (468 cells, from 4 mice). (**e**) *In vivo* right PN “optrode” recording setup. (**f**,**g**) Multi-unit activity in the right PN from a rat injected with pAAV2/9-hSyn-ChR2-mCherry in response to trains of 7 light pulses (470 nm, 10 mW/mm^2^, 20 Hz, 15 ms pulse duration). Blue bars represent light on. (**h**) Schematic illustration of *in vivo* optical stimulation and LFP recording in the left MCP. (**i**) Example of ChR2-mCherry expression in the left MCP. White dashed line: optrode position. (**j**,**k**) Trains of 7 light pulses (470 nm, 25 mW/mm^2^, 20 Hz, 15 ms pulse duration) also evoked robust LFP responses in the left MCP of a wake behaving rats. Note that the graph. (**j**) Illustrates an example of the mean value of 100 light-induced LFPs. Data are represented as mean ± s.e.m.

To address this question, we injected rats with pAAV 2/9-hSyn-ChR2-mCherry or pAAV 2/9-hSyn-mCherry virus into the right PN (Fig. 1a,b). Three–four weeks after virus injection, the expression of ChR2 was confirmed by immunohistochemistry. 93.49 ± 0.74% of NeuN-immunopositive cells expressed ChR2-mcherry and the promoter also provided high specificity near virus injection sites, because all of ChR2-mcherry-expressing cells were NeuN-immunopositive cells (Fig. 1c,d). To assess functionality of ChR2, we performed optrode recordings in anesthetized rats and confirmed that ChR2-expressing cells showed robust responses to light stimulation (470 nm, 350 ms, 10 mW/mm^2^, 20 Hz, 15 ms pulse duration; Fig. 1e–g). Moreover, as expected, LED illumination (470 nm, 350 ms, 25 mW/mm^2^, 20 Hz, 15 ms pulse duration; Fig. 1h–k) of the ChR2-expressing mossy fibers also showed robust LFP responses in awake behaving rats.

**Figure 2:**
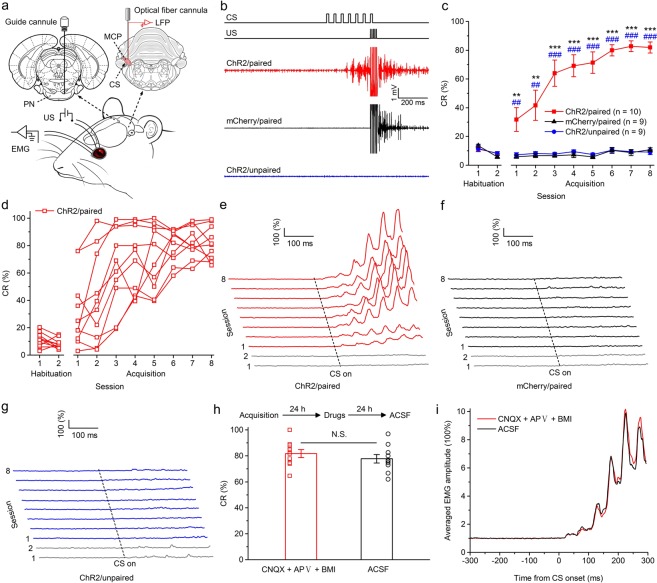
Optogenetic stimulation of mossy fibers in the left MCP as a CS is sufficient for the acquisition of the DEC. (**a**) Behavioral diagram. Rats with virus injection were implanted with 4 electrodes into the upper left eyelid for delivery of the US and for recording the EMG activity of the left O.O. muscle. Moreover, an optrode was targeted into the left MCP for optical stimulation and recording LFP. A guide cannula was implanted into the right PN for drug injection. (**b**) Upper panel: the delay conditioning paradigm illustrating the timing of the CS and US. Below panel: the representative O.O. EMG of the ChR2/paired, mCherry/paired, and ChR2/unpaired groups on the 8th conditioning session. (**c**) Average CR% for the ChR2/paired, mCherry/paired, and ChR2/unpaired groups (* and ^#^ indicate significant differences between the ChR2/paired group and mCherry/paired and the ChR2/unpaired groups; ** or ^##^*P* < 0.01, *** or ^###^*P* < 0.001; 2-way ANOVA with repeated measures followed by Tukey post hoc test). (**d**) Individual learning curves of rats in ChR2/paired group. (**e**–**g**) EMG response topographies across two habituation and eight acquisition training sessions in ChR2/paired (**e**), mCherry/paired (**f**), and ChR2/unpaired (**g**) groups. (**h**) CNQX, APV, and BMI administration did not affect the CR% of ten learned rats (N.S., not significant, 2-tailed paired Student’s t-test). (**i**) The EMG response topographies for CNQX, APV, and BMI infusion or ACSF infusion were shown. Data are represented as mean ± s.e.m.

Three–four weeks after virus injection, the rats were implanted with an optrode consisting of a fiber optic cannula with two recording electrodes (insulated stainless steel wires, 76.2 μm inner diameter) directly attached to the optical fiber (200 μm core diameter, 0.39 numerical aperture) targeting the left MCP. Moreover, a guide cannula was implanted into the right PN for drug infusion (Fig. 2a). One week after surgery, some rats expressing ChR2 (ChR2/paired group) and some rats expressing mCherry (mCherry/paired group) were conditioned by using a delay paradigm in which they received paired presentations of the optogenetic CS (470 nm, 350 ms, 25 mW/mm^2^, 20 Hz, 15 ms pulse duration) and periorbital shock US (Fig. 2a,b). Furthermore, another group of rats expressing ChR2 (ChR2/unpaired group) received random presentations of the same CS and US, which were explicitly unpaired in time.

The rats of the three groups showed low frequency of spontaneous eyeblinks, which did not differ significantly from each other during two habituation sessions (*F*_(2,25)_ = 0.002, *P* = 0.998; Fig. 2b–g). In contrast, there was a progressive increase in the CR%, reaching an asymptotic level, in the ChR2/paired group across acquisition sessions 1–8, which was due to associative learning, as the ChR2/unpaired and mCherry/paired groups did not show increases in responding across acquisition sessions 1–8 (Fig. 2b–g). Moreover, all of 10 rats in ChR2/paired group reached the learning criterion of 6 consecutive CRs in a single session (Fig. 2d). These were confirmed by a two-way repeated measures ANOVA. There was a significant interaction between groups and sessions (*F*_(14,175)_ = 12.786, *P* < 0.001) and significant effects of group (*F*_(2,25)_ = 79.981, *P* < 0.001) and of session (*F*_(7,175)_ = 17.215, *P* < 0.001). Post-hoc tests indicated that rats in the ChR2/paired group showed more CRs than rats in the ChR2/unpaired and mCherry/paired groups on sessions 1–8 (all *P* < 0.01), which, however, did not differ significantly from each other (all *P* > 0.05; Fig. 2c). In addition, there was a progressive increase in the CR peak amplitude of ChR2/paired (learned) rats (Supplementary Fig. S1a,b).

To further preclude contributions to DEC learning from the extra-cerebellar regions, we injected the learned (acquired) rats with AMPA receptor antagonist CNQX, NMDA receptor antagonist APV and GABA_A_ receptor antagonist BMI into the PN to reversibly block glutamatergic and GABAergic inputs to the PN from the extra-cerebellar and cerebellar regions. As expected, drug infusion had no significant effect on the CR% compared with ACSF infusion (*t*_(18)_ = 0.895, *P* = 0.383; Fig. 2h,i). Together, these data suggest that direct optogenetic stimulation of mossy fibers in the MCP is a very effective and sufficient CS for establishing DEC in rats, and that the cerebellum has the ability to support the simple DEC without any modulation by the extracerebella regions.

### The optogenetic CS supported the acquisition of TEC with a 150-ms trace interval in five out of seven rats

**Figure 3:**
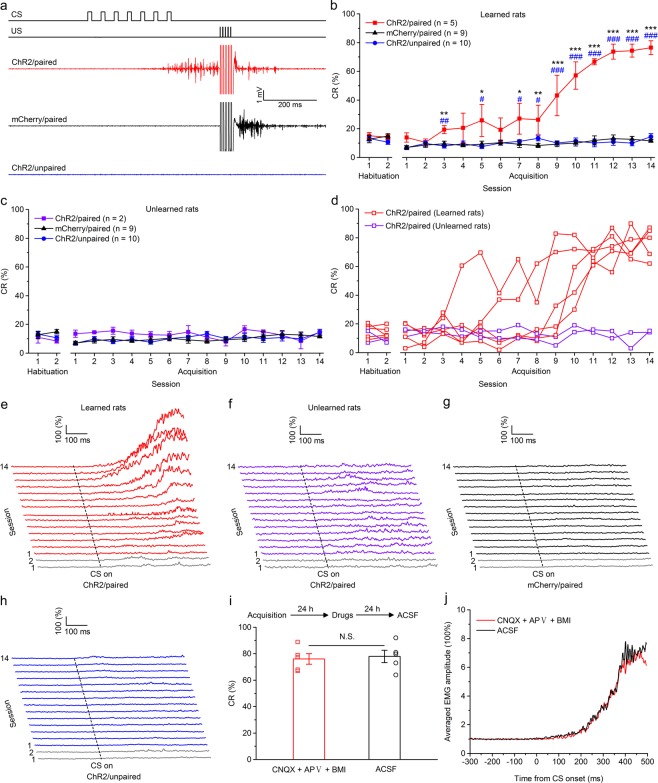
Optogenetic stimulation of mossy fibers in the left MCP as a CS supports the acquisition of the TEC with a 150-ms trace interval. (**a**) Upper panel: the trace conditioning with a 150-ms trace interval paradigm illustrating the timing of the CS and US. Below panel: the representative O.O. EMG of the ChR2/paired, mCherry/paired, and ChR2/unpaired groups on the 14th conditioning session. (**b**,**c**) Average CR% for learned rats (**b**) and unlearned rats (**c**) in the ChR2/paired group, and all rats in mCherry/paired and ChR2/unpaired groups (* and ^#^ indicate significant differences between the ChR2/paired group and mCherry/paired and the ChR2/unpaired groups; ** or ^##^*P* < 0.01, *** or ^###^*P* < 0.001; 2-way ANOVA with repeated measures followed by Tukey post hoc test). (**d**) Individual learning curves of learned rats (red) and unlearned rats (violet) in ChR2/paired group. (**e**–**h**) EMG response topographies across two habituation and fourteen acquisition training sessions for learned rats (**e**) and unlearned rats (**f**) in the ChR2/paired group, and all rats in mCherry/paired (**g**) and ChR2/unpaired (**h**) groups. (**i**) CNQX, APV, and BMI administration did not affect the CR% of five learned rats (N.S., not significant, 2-tailed paired Student’s t-test). (**j**) The EMG response topographies for CNQX, APV, and BMI infusion or ACSF infusion were shown. Data are represented as mean ± s.e.m.

Next, we tested whether optogenetic stimulation of mossy fibers in the MCP is a sufficient CS to support the acquisition of TEC with a trace interval of 150 ms. Rats in this experiment were injected with the viruses and underwent identical surgery as the above mentioned three groups, then trained with paired or unpaired trace paradigm with a trace interval of 150 ms. As expected, the rats of the three groups also showed low frequency of spontaneous eyeblinks, which did not differ significantly from each other during two habituation sessions (*F*_(2,21)_ = 0.668, *P* = 0.523 and *F*_(2,18)_ = 0.730, *P* = 0.496, respectively; Fig. 3b,c). However, most of (five out of seven) rats in ChR2/paired group received paired training with mossy fibers optogenetic stimulation acquired the TEC with a 150-ms trace interval and reached the learning criterion of 6 consecutive CRs in a single session, whereas all rats of the ChR2/unpaired and mCherry/paired groups did not (Fig. 3a–h). A two-way repeated measures ANOVA comparing the CR% among learned rats of ChR2/paired, ChR2/unpaired and mCherry/paired groups revealed that there was a significant interaction between groups and sessions (*F*_(26,273)_ = 18.813, *P* < 0.001) and significant effects of both group (*F*_(2,21)_ = 50.080, *P* < 0.001) and session (*F*_(13,273)_ = 30.988, *P* < 0.001). Post-hoc tests showed that the ChR2/paired group produced a significantly greater CR% than the ChR2/unpaired and mCherry/paired groups on sessions 3, 5, 7–14 (all *P* < 0.05), which, however, did not differ significantly from each other (all *P* > 0.05; Fig. 3b). In addition, there was a progressive increase in the CR peak amplitude of ChR2/paired (learned) rats (Supplementary Fig. S2a,b). However, a two-way repeated measures ANOVA comparing the CR% among unlearned rats of ChR2/paired group, ChR2/unpaired and mCherry/paired groups revealed that there was no significant interaction between groups and sessions (*F*_(26,234)_ = 0.706, *P* = 0.855), and no significant effects of group (*F*_(2,18)_ = 1.130, *P* = 0.345) or of session (*F*_(13,234)_ = 0.762, *P* = 0.700; Fig. 3c).

Similarly, pharmacological blocking glutamatergic and GABAergic inputs to the PN (then to cerebellum) from the extra-cerebellar and cerebellar regions had no significant effect on the CR% compared with ACSF infusion (*t*_(8)_ = 0.303, *P* = 0.769; Fig. 3i,j). Together, these data suggest that optogenetic stimulation of mossy fibers in the MCP is an effective CS for most rats to establish TEC with a 150-ms trace interval.

### The optogenetic CS supported the acquisition of TEC with a 350-ms trace interval in three out of nine rats

**Figure 4:**
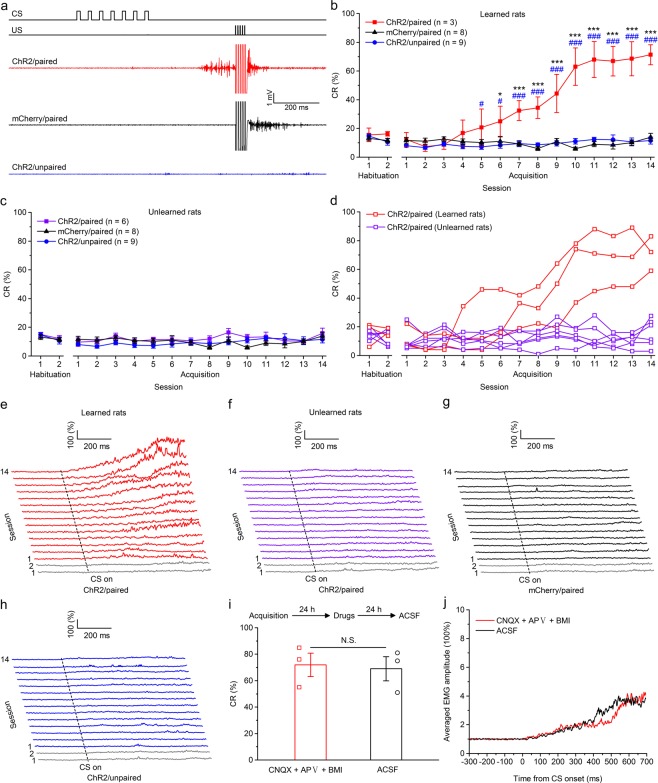
Optogenetic stimulation of mossy fibers in the left MCP as a CS supports the acquisition of the TEC with a 350-ms trace interval. (**a**) Upper panel: the trace conditioning with a 350-ms trace interval paradigm illustrating the timing of the CS and US. Below panel: the representative O.O. EMG of the ChR2/paired, mCherry/paired, and ChR2/unpaired groups on the 14th conditioning session. (**b**,**c**) Average CR% for learned rats (**b**) and unlearned rats (**c**) in the ChR2/paired group, and all rats in mCherry/paired and ChR2/unpaired groups (* and ^#^ indicate significant differences between the ChR2/paired group and mCherry/paired and the ChR2/unpaired groups; ** or ^##^*P* < 0.01, *** or ^###^*P* < 0.001; 2-way ANOVA with repeated measures followed by Tukey post hoc test). (**d**) Individual learning curves of learned rats (red) and unlearned rats (violet) in ChR2/paired group. (**e**–**h**) EMG response topographies across two habituation and fourteen acquisition training sessions for learned rats (**e**) and unlearned rats (**f**) in the ChR2/paired group, and all rats in mCherry/paired (**g**) and ChR2/unpaired (**h**) groups. (**i**) CNQX, APV, and BMI administration did not affect the CR% of three learned rats (N.S., not significant, 2-tailed paired Student’s t-test). (**j**) The EMG response topographies for CNQX, APV, and BMI infusion or ACSF infusion were shown. Data are represented as mean ± s.e.m.

We further tested whether optogenetic stimulation of mossy fibers in the MCP is a sufficient CS to support the acquisition of TEC with a trace interval of 350 ms. Rats in this experiment were injected with the viruses and underwent identical surgery as the above mentioned three groups, then trained with paired or unpaired trace paradigm with a trace interval of 350 ms. As expected, the rats of the three groups also showed low frequency of spontaneous eyeblinks, which did not differ significantly from each other during two habituation sessions (*F*_(2,17)_ = 0.829, *P* = 0.453 and *F*_(2,20)_ = 0.275, *P* = 0.762, respectively; Fig. 4b,c). However, a few rats (three out of nine) of ChR2/paired group received paired training with mossy fibers optogenetic stimulation acquired the TEC with a 350-ms trace interval, and reached the learning criterion of 6 consecutive CRs in a single session, whereas all rats of the ChR2/unpaired and mCherry/paired groups did not (Fig. 4a–h). A two-way repeated measures ANOVA comparing the CR% among learned rats of ChR2/paired, ChR2/unpaired and mCherry/paired groups revealed that there was a significant interaction between groups and sessions (*F*_(26,221)_ = 19.556, *P* < 0.001) and significant effects of both group (*F*_(2,17)_ = 38.311, *P* < 0.001) and session (*F*_(13,221)_ = 30.059, *P* < 0.001). Post-hoc tests showed that the ChR2/paired group produced a significantly greater CR% than the ChR2/unpaired and mCherry/paired groups on sessions 5–14 (all *P* < 0.05) and on sessions 6–14 (all *P* < 0.05), respectively, which, however, did not differ significantly from each other (all *P* > 0.05; Fig. 4b). In addition, there was a progressive increase in the CR peak amplitude of ChR2/paired (learned) rats (Supplementary Fig. S1a,b). However, a two-way repeated measures ANOVA comparing the CR% among unlearned rats of ChR2/paired group, ChR2/unpaired and mCherry/paired revealed that there was no significant interaction between groups and sessions (*F*_(26,260)_ = 1.120, *P* = 0.318), and no significant effects of group (*F*_(2,20)_ = 1.085, *P* = 0.357) or of session (*F*_(13,260)_ = 1.581, *P* = 0.090; Fig. 4c).

Similarly, pharmacological blocking glutamatergic and GABAergic inputs to the PN (then to cerebellum) from the extra-cerebellar and cerebellar regions had no significant effect on the CR% compared with ACSF infusion (*t*_(4)_ = 0.230, *P* = 0.829; Fig. 4i,j). Taken together, these data suggest that optogenetic stimulation of mossy fibers in the MCP is an effective CS for a few rats, but not for most rats, to establish TEC with a 350-ms trace interval.

### The optogenetic CS failed to support the acquisition of TEC with a 500-ms trace interval

**Figure 5:**
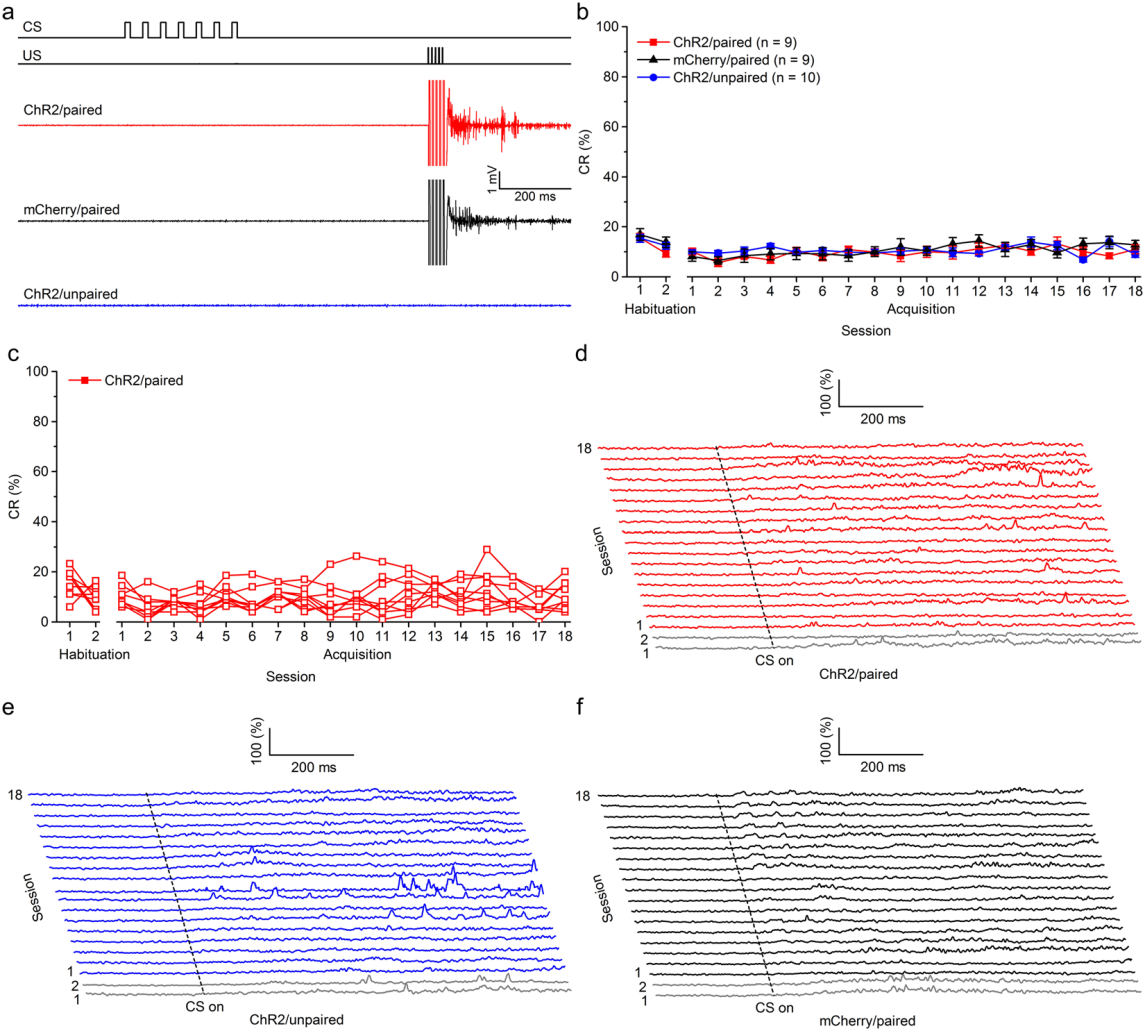
Optogenetic stimulation of mossy fibers in the left MCP as a CS fails to support the acquisition of the TEC with a 500-ms trace interval. (**a**) Upper panel: the trace conditioning with a 500-ms trace interval paradigm illustrating the timing of the CS and US. Below panel: the representative O.O. EMG of the ChR2/paired, mCherry/paired, and ChR2/unpaired groups on the 18th conditioning session. (**b**) Average CR% for ChR2/paired, mCherry/paired and ChR2/unpaired groups (2-way ANOVA with repeated measures). (**c**) Individual learning curves of rats in ChR2/paired group. (**d**–**f**) EMG response topographies across two habituation and eighteen acquisition training sessions in the ChR2/paired (**d**), mCherry/paired (**e**), and ChR2/unpaired (**f**) groups. Data are represented as mean ± s.e.m.

Finally, we examined whether optogenetic stimulation of mossy fibers in the MCP is a sufficient CS to support the acquisition of TEC with a trace interval of 500 ms. Rats in this experiment were injected with the viruses and underwent identical surgery as the above mentioned three groups, then trained with paired or unpaired trace paradigm with a trace interval of 500 ms. As expected, the rats of the three groups also showed low frequency of spontaneous eyeblinks, which did not differ significantly from each other during two habituation sessions (*F*_(2,25)_ = 1.046, *P* = 0.366; Fig. 5b). Moreover, all rats of ChR2/paired group, received paired training with mossy fibers optogenetic stimulation failed to acquire the TEC with a 500-ms trace interval and did not reach the learning criterion of 6 consecutive CRs in a single session. Furthermore, all rats of the ChR2/unpaired and mCherry/paired groups also failed to acquire the TEC with a 500-ms trace interval and meet the learning criterion (Fig. 5b–f). These were confirmed by a two-way repeated measures ANOVA. There was a significant interaction between groups and sessions (*F*_(34,425)_ = 4.834, *P* = 0.004) and significant effects of session (*F*_(17,425)_ = 2.704, *P* < 0.001), but there was no significant effect of group (*F*_(2,25)_ = 0.175, *P* = 0.840).

**Figure 6:**
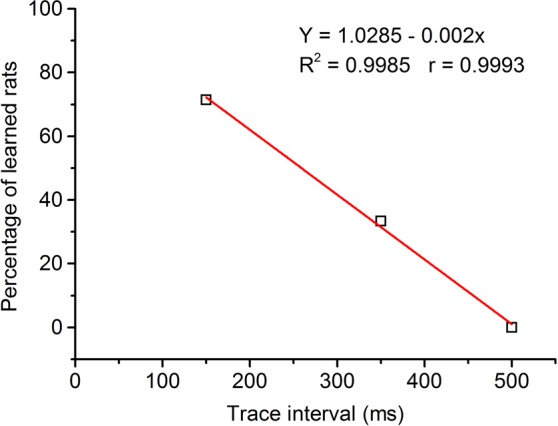
Regression analysis of the relationships between percentage of conditioned responses and length of race interval. Percentage of learned rats correlates well with reduction in length of the trace interval. Best fit line indicates a significant negative correlation (R^2^ = 0.9985, *P* = 0.024).

As illustrated in Fig. 6, the percentage of learned rats was linearly related to the trace interval (slope, −0.002). The regression line confirmed a significant negative correlation between the percentage of learned rats and the length of the trace interval (R^2^ = 0.9985, *P* = 0.024). Together, these findings suggest that optogenetic stimulation of mossy fibers in the MCP is a sufficient CS to support the acquisition of DEC and TEC with a trace interval of 150 and 350 ms, but usually do not support the acquisition of TEC with a trace interval of 500 ms.

## Discussion

In this study, we examined the ability of the cerebellum to independently learn the DEC and TEC with various trace intervals using pharmacological and optogenetic approaches in awake, freely moving rats. First, we showed that optogenetic stimulation of mossy fibers in the MCP as a CS paired with a peripheral US is sufficient to establish DEC in rats, and that blocking the possible inputs to the PN has no significant effect on the DEC. In contrast, rats given unpaired presentations of the optogenetic CS and peripheral US in ChR2/unpaired group showed no increase in eyeblink responses across 8 training sessions, indicating that CRs observed in the ChR2/paired group were due to associative learning. Combined with previous studies, our present results provide compelling evidence that the cerebellum comprises essential and sufficient sites to support the simple DEC. Given that a various brain areas within the forebrain and midbrain (e.g., mPFC, hippocampus, amygdala, medial auditory thalamus, inferior colliculus) have been verified to play an important role in the DEC under suboptimal, or even under optimal learning conditions^17,90,91,97–100^, the present study suggests that extra-cerebellar regions maybe contribute to facilitate but not modulate the simple DEC. Second, we showed that optogenetic stimulation of mossy fibers in the MCP is a sufficient CS for some rats to acquire the TEC with a trace interval of 150 or 350 ms, but not of 500 ms. However, rats given unpaired presentations of the optogenetic CS and peripheral US in ChR2/unpaired group also showed no increase in eyeblink responses across the 14 or 18 training sessions.

Kalmbach *et al*. reported that direct electrical stimulation of mossy fibers in the MCP as the CS could support the DEC and TEC with a trace interval of 200, 300, or 400 ms, but failed to support the TEC with a trace interval of 500 ms^25^. It is important to note that in their study all of six rabbits reached the learning criterion of the DEC or TEC with a trace interval of 200, 300, or 400 ms. However, in the present study only part of rats learned the TEC with a trace interval of 150 or 350 ms when the CS was the optogenetic stimulation of mossy fibers in the MCP. The difference was not due to the inaccurate placements of the optrode, because we have examined all the placements of the optrode and data from animals were discarded in the analysis if the placements of the optrode were not in the MCP. Moreover, the difference was not attributable to the insufficient optogenetic stimulation either, because the amplitude of LFP evoked by the optogenetic stimulation in the MCP was not significant lower in unlearned rats compared with that in learned rats (data not shown). In addition, all rats successfully acquired the DEC when used the optogenetic CS in the present study. Therefore, our results indicate that some rats, but not all, have the ability to learn TEC with a trace interval of 150 or 350 ms due to the individual differences. In fact, the individual difference has also been shown in TEC with a 500-ms trace interval using the peripheral CS in previous studies. For example, we have found that about 15% of rats trained with a tone CS paired with a periorbital US failed to acquire the TEC with a 500-ms trace interval. Moreover, Oswald *et al*. have reported that 10–15% of rabbits trained with a tone CS paired with an airpuff US failed to meet the learning criterion of the TEC with a 500-ms trace interval^76^. Together, it could be attributed to electrical current diffusion that why all of six rabbits acquiring the TEC with a trace interval of 200, 300, or 400 ms in the previous study, as the previous study did not block the possible inputs form extra-cerebellar regions to the PN. The diffusion of electrical current might stimulate many regions such as the meninges, leading to a somatosensory CS, which has been proven to be an effective and sufficient CS for EBC^12,87^.

Although some of the rats successfully acquired the TEC with a trace interval of 150 or 350 ms in the present study, they learned more slowly with the optogenetic stimulation CS compared with the auditory (tone) or visual (light) CS in our previous studies. In the 150-ms trace paradigm, only one rats learned in the earlier sessions while most rats (four rats) learned in the later sessions and the mean percentage of CR increased slowly across the 14 training sessions, yielding a slower and flatter learning curve (Fig. 3d). Similarly, the phenomenon was also showed in the 350-ms trace paradigm, in which all of three learned rats acquired later (Fig. 4d). It seems that in some rats the cerebellum has the necessary ability to support the slowly acquisition of TEC with a trace interval of 150 or 350 ms. However, without the modulation and facilitation effects from extra-cerebellar regions, more training was required for the TEC acquisition.

In fact, the role of extra-cerebellar regions (e.g., mPFC and hippocampus) in the TEC has been widely examined^81–83^. Several lines of indirect evidence suggests that successful learning of TEC with the 500-ms or longer trace interval required the involvement of extra-cerebellar regions (e.g., mPFC and hippocampus)^92,101–105^. Weiss and Disterhoft^106^ first proposed that forebrain structures, such as the mPFC, may act to facilitate pontine inputs to the cerebellum to modulate TEC with a 500 ms trace interval in rabbits. The persistent activity in the mPFC existed during the 500-ms trace interval in the TEC supported this assumption^105,107,108^. In addition, our previous study showed that optogenetic inhibition of the mPFC or its axon terminates at the pontine nuclei during the trace interval, but not during the CS or intertrial interval period, significantly disrupt the TEC with a 500 ms trace interval in rats. Thus, combined with these previous studies, our present results suggest that the cerebellum maybe is unable to independently support the acquisition of TEC with a trace interval of 500 ms.

It is worth noting that each posterior single pulse of 7 light pulse trains applied at the mossy fibers in the MCP evoked orbicularis oculi muscle corresponding activity during the DEC training (Fig. 4b,e). The orbicularis oculi muscle activity may be a kind of learned (conditioned) response. Because this phenomenon has not been observed before the rats learned in ChR2/paired and ChR2/unpaired groups (Fig. 4b,e,g). Moreover, this orbicularis oculi muscle activity usually clearly presented after the first two pulses of the 7 light pulse trains (Fig. 4b,e). It should also be noted that there was a major difference between the present study and the result reported by Gruart *et al*.^67^ and Jiménez-Díaz *et al*.^68^. The orbicularis oculi muscle activity evoked by a train (20 Hz) of electrical pulses applied to the posterior interpositus nucleus was an unlearned (unconditioned) response in those studies.

In conclusion, our findings clearly indicate that the cerebellum has the ability to independently support the simple DEC, and the TEC with a trace interval of 150 or 350 ms, but it is unable for the cerebellum networks to establish TEC with a trace interval of 500 ms or longer without the involvement of extra-cerebellar regions. The present results advance our understanding of the mechanism and ability of cerebellum in associative motor learning.

## Methods

### Animals

Adult male Sprague Dawley (SD) rats, weighing 350–400 g (3–4 months) at the time of virus injection, were individually housed in standard cages on a 12:12 light/dark cycle with free access to food and water *ad libitum*. Behavioral experiments were performed during the light cycle. The room temperature was maintained at 22 ± 1 °C. All animal procedures were approved by the Animal Care Committee of the Army Medical University and were performed in accordance with the principles outlined in the National Institutes of Health Guide for the Care and Use of Laboratory Animals.

### Virus Injection

Rats were anaesthetized with a mixture of ketamine (100 mg/kg, i.p., Gutian, Fujian, China) and xylazine (9 mg/kg, i.p., Sigma-Aldrich, St. Louis, MO, USA) and fixed in a stereotaxic apparatus (Model 942, David Kopf Instruments, Tujunga, California, USA) as described previously^17,18,109^. The virus was injected using a glass micropipette (tip diameter 10–20 µm) attached to a 5 µl Hamilton microsyringe (51189, Stoelting, Wood Dale, Illinois, USA). The injection rate (0.05 µl/min) was controlled by a stereotaxic microsyringe pump (53311, Stoelting). After injection, the needle was left in place for 5 additional minutes and then slowly withdrawn. Rats were unilaterally microinjected with 0.5 µl of pAAV 2/9-hSyn-ChR2(H134R)-mCherry (Virus titres: 5.18 × 10^12^ GC/ml) or pAAV 2/9-hSyn-mCherry (Virus titers: 7.04 × 10^12^ GC/ml) into the right PN [anteroposterior (AP) −7.32 mm, mediolateral (ML) 0.9 mm, dorsoventral (DV) −10.0 mm; i.e., contralateral to the training eye; Fig. 1a,b]. These above vectors were obtained from AddGene and packaged by Obio Technology (Shanghai, China).

### Immunohistochemistry

For immunohistochemistry experiments, rats were injected with an overdose of 10% chloral hydrate (1000 mg/kg, i.p., Kelong, Chengdu, China) and perfused transcardiacally with physiological saline followed by cold 4% paraformaldehyde (PFA; prepared in 0.1 M of phosphate buffer, pH 7.4). The brains were removed from the skull and stored in 4% PFA at 4 °C for 24 h, then transferred to a 30% sucrose solution at 4 °C for 48 h. 30 μm-thick coronal sections were cut on a freezing microtome (CM3050 S, Leica, Germany) and collected in cold phosphate buffer saline (PBS, 0.01 M, pH 7.4). For immunostaining, each slice was placed in PBST (PBS + 0.3% Triton X-100) with 2% normal bovine serum albumin for 1 h then incubated with primary antibody at 4 °C for 24 h (Mouse Anti-NeuN 1:500, PC213L, Merck). Slices then underwent three wash steps for 10 min each in PBST, followed by 2 h incubation with secondary antibody (Goat anti-mouse conjugated to AlexaFluor488, 1:500, Invitrogen). Slices were washed with PBST (once, 10 min) and incubated for 10 min with Hoechst (1:2000, 861405, Sigma-Aldrich), and then underwent three more wash steps of 10 min each in PBST, followed by mounting and coverslipping on microscope slides. Confocal fluorescence images were acquired on a Carl Zeiss LSM 780 scanning laser microscope (Germany) using a 10 × air objective or a 40 × oil immersion objective.

### Electrophysiological Verification of Optogenetics

3–4 weeks after virus injection, rats were anaesthetized by 10% chloral hydrate (400 mg/kg, i.p., Kelong) and their heads were placed in a stereotaxic apparatus (Model 942, David Kopf). An optrode (optrode A) consisting of a fiber optic cannula (ceramic ferrule: diameter 1.25 mm; optical fiber: 200 μm core diameter, 0.39 NA, FT200EMT, Thorlabs, Newton, New Jersey, USA) with a multi-wire electrode tightly coupled with an optical fiber, with the tips of the electrodes extending approximately 300 μm beyond the tip of optical fiber was used for LED illumination and extracellular recordings. Electrodes were made of 16 individually insulated nichrome wires (17.78 μm inner diameter, 761000, A-M Systems, Sequim, WA, USA), attached to a 20-pin connector.

To confirm the physiological effect of LED illumination of ChR2 on PN neuronal activities, the optrode (optrode A, as described above) was slowly lowered to the right PN using a micromanipulator (IVM-1000, Scientifica, UK) in anesthetized rats. The optical fiber was connected to a 470-nm LED (M470F1, Thorlabs, Newton, New Jersey, USA) controlled by a pulse stimulator (Master-9, A.M.P.I., Jerusalem, Israel; Fig. 1e). After light-responsive cells were detected, a series of light stimuli were conducted: 100 epochs of 350-ms light pulse trains (470 nm, 10 mW/mm^2^, 20 Hz, 15 ms pulse duration), separated by a variable interval of 20–40 s (with a mean of 30 s). Extracellular signals were bandpass filtered (0.3–5 kHz and 0.3–500 Hz, respectively), amplified (1000×) using a 16-channel microelectrode amplifier (model 3600, A-M Systems, Sequim, WA, USA) and acquired with a data acquisition system (Powerlab 16/35, ADInstuments, New South Wales, Australia) with a sampling rate of 20 kHz. Spike data were analyzed with NeuroExplorer 4 (MicroBrightField, Williston, VT, USA), a neurophysiological data analysis software.

In addition, to confirm the physiological effect of LED illumination of ChR2 on mossy fibers activities, the other type of optrode (optrode B; ceramic ferrule: diameter 1.25 mm; optical fiber: 200 μm core diameter, 0.39 NA, FT200EMT) consisting of a fiber optic cannula with two recording electrodes (insulated stainless steel wires, 76.2 μm inner diameter, 790900, A-M Systems) directly attached to the optical fiber with the same location of their tips was used for simultaneous LED illumination (470 nm, 25 mW/mm^2^, 20 Hz, 15 ms pulse duration) and for local field potential (LFP) recording in left MCP of each awake behaving rat (as described below; Figs 1h and 2a). The LFP signals were bandpass filtered (0.3–500 Hz), amplified (1000×) using a 16-channel differential amplifier (model 3500, A-M Systems) and acquired with a data acquisition system (Powerlab 16/35) during behavior training.

### Surgery

All of the rats used for behavior training were anaesthetized with a mixture of ketamine (100 mg/kg, i.p., Gutian, Fujian, China) and xylazine (9 mg/kg, i.p., Sigma-Aldrich) and fixed in a stereotaxic apparatus (Model 942, David Kopf) after virus expression. For delivering the shock US and recording the differential electromyography (EMG) activity of the ipsilateral orbicularis oculi muscle, rats were implanted with four electrodes, made of insulated stainless steel wires (76.2 μm inner diameter, 790900, A-M Systems) in the upper eyelid of the left eye. One pair of electrodes for delivering the shock US was implanted into subdermal caudal to the left eye. The second pair of electrodes was implanted into the ipsilateral orbicularis oculi muscle to record its EMG activity. The electrode tips were bent as a hook to facilitate a stable insertion in the upper eyelid (Fig. 2a). Moreover, a bare silver wire (0.1 mm in diameter) was connected to four stainless steel skull screws as a ground. In addition, for optogenetic stimulation of the left MCP, an optrode (optrode B, as described above) were lowered to 0.3 mm over the left MCP (AP −9.7 mm, ML 3.5 mm, DV −6.4 mm; Fig. 2a). For drug blocking the possible inputs from extra-cerebellar regions into the cerebellum, one guide cannula was implanted into the right PN (AP −9.7 mm, ML 3.5 mm, DV −6.1 mm mm). Since the tip of the infusion cannula extended 0.3 mm beyond the tip of the guide cannula, the final infusion positions were at following stereotaxic coordinates: AP −9.7 mm, ML 3.5 mm, DV −6.4 mm mm (Fig. 2a). The wires were connected to an eight-pin mini-strip connector. The mini-strip connector, optrode, and guide cannula were cemented to the skull with dental cement. After the surgery, the animals were allowed 1 week of recovery.

### Behavioral Procedures

Prior to acquisition training, all rats underwent two initial 50 min habituation sessions in a plastic box (35 × 25 × 20 cm), housed within a sound- and light-attenuating chamber. For providing a baseline spontaneous eyeblink rate, the EMG activity of the orbicularis oculi muscle of each rat was recorded for two habituation sessions in the same way as the conditioning session, except that no stimuli were presented (see below).

The eyeblink conditioning was carried out using a delay or trace paradigm. The CS was a 350-ms optogenetic stimulation (470 nm, 25 mW/mm^2^, 20 Hz, 15 ms pulse duration) of the left MCP, which was delivered from a 470-nm LED (M470F1) controlled by a pulse stimulator (Master-9). The US was a 50-ms periorbital electrical shock (100 Hz, 1 ms pulse duration, square, cathodal pulse), delivered from a stimulus isolator (ISO-Flex, A.M.P.I., Jerusalem, Israel), controlled by a pulse stimulator (Master-9). The intensity of the shock US was carefully calibrated to give the minimal current required to elicit a discrete eyeblink response (1–2 mA). The US intensity was set before the first acquisition session and was not changed during the rest of the experiment. The daily conditioning session (day) consisted of ten 10-trial blocks, each of which comprised nine CS-US paired trials and one CS-alone trial. The trials were separated by a variable inter-trial interval of 20–40 s (with a mean of 30 s). During the CS–US paired delay conditioning trials, the CS terminated simultaneously with the US (Fig. 2b). During the CS–US paired trace conditioning trials, the US started 150 (Fig. 3a), 350 (Fig. 4a), or 500 ms (i.e., the trace interval was 150, 350, or 500 ms; Fig. 5a) after the termination of the CS. The ChR2/unpaired group received the same CS and US, but the US was presented with a random interval between 1 and 10 s after the CS onset.

### Drug Delivery

For blocking the possible inputs from extra-cerebellar regions into the cerebellum, the α-amino-3-hydroxy-5-methyl-4-isoxazolepropionic acid (AMPA) receptor antagonist 6-Cyano-7-nitroquinoxaline-2,3-dione disodium salt hydrate (CNQX, C239, Sigma-Aldrich), N-Methyl-D-aspartate (NMDA) receptor antagonist DL-2-amino-5-phosphonovaleric acid (APV, A5282, Sigma-Aldrich), and the γ-aminobutyric acid type A (GABA_A_) receptor antagonist bicuculline methiodide (BMI, ab120108, Abcam) were dissolved in artificial cerebrospinal fluid (ACSF) consisting of (in mM): 126 NaCl, 5 KCl, 1.25 NaH_2_PO_4_, 2 MgSO_4_, 26 NaHCO_3_, 2 CaCl_2_, and 10 glucose (pH 7.35–7.40). Twenty-four hours after acquisition training, the rats were injected with 1.0 μl of CNQX (3.0 mM), APV (50.0 mM) and BMI (11 nM) mixed liquor into the right PN of the conditioned rats 30 min before the first test training^6,110,111^. Moreover, these rats were also injected with 1.0 μl of ACSF into the right PN 30 min before the second test training 24 h later (e.g., Fig. 2h). Infusion procedures for each animal included removal of the internal stylet from the guide cannula, insertion of a stainless steel infusion cannula that extended 0.5 mm beyond the tip of the guide cannula, infusion of the solutions at a constant rate of 0.1 µl/min, removal of the infusion cannula 5 min after the cessation of infusion, and finally reinsertion of the internal stylet^97,109^. The constant injection rate was maintained using a microsyringe pump (53222 V, Stoelting).

### Behavioral Data Analysis

EMG activity of the orbicularis oculi muscle and the LFP signals were band-pass filtered (0.1–3 kHz) using a 16-channel differential amplifier (model 3500, A-M Systems) and acquired with a data acquisition system (Powerlab 16/35, ADInstuments) with a sampling rate of 10 kHz.

EMG data were analyzed off-line for quantification of CRs with the help of a home-made program. The collected EMG data were full-wave rectified and integrated with a 1-ms time constant. The integrated EMG activity was then calculated to the standard score compared to the mean of the baseline activity for the 0–300 ms before the CS onset in each trial. Thus, the EMG amplitude is given as a percentage of the baseline (100%) averaged EMG amplitude. The mean plus 5 times standard deviation (SD) of standard EMG activity during the baseline period of each trial was defined as the trial threshold. If the standard EMG amplitude during baseline period exceeded the trial threshold and lasted ≥ 5 ms, the trial was regarded as a hyperactivity trial and excluded from further analysis. Moreover, a CS–US paired trial was considered to contain the CR if the standard EMG amplitudes exceeded the trial threshold and lasted ≥ 5 ms during the period of 0–220 ms before the US onset (i.e., CR period). In the CS-alone trials, the CR period was extended the end of the expected US period. For unpaired training rats, the CR-like period was analyzed for four 220-ms periods, which correspond to the CR periods analyzed in the CS-alone trials of one delay and three trace paradigms. The percentage of CR (CR%) was defined as the ratio of the number of trials containing the CR to the total number of valid trials. The CR peak amplitude was defined as the maximum amplitude change from the baseline during the CR period. Note that only trials containing CRs were selected for analysis of CR peak amplitude. The CR peak amplitude for the unlearned rats were not further analyzed since so few responses were seen. Moreover, the “averaged EMG amplitude” was analyzed by averaging EMG of individual rat over the valid trials and further averaged for each group. The learning (acquisition) criterion was at least 6 consecutive CRs with one acquisition training session^17,18,66,112,113^.

### Histology

After behavioral experiments, rats were deeply anaesthetized with an overdose of 10% chloral hydrate (1000 mg/kg, i.p., Kelong) and perfused transcardiacally with physiological saline followed by cold 4% paraformaldehyde (PFA; prepared in 0.1 M of phosphate buffer, pH 7.4). The brains were removed from the skull and stored in 4% PFA at 4 °C for 24 h, then transferred to a 30% sucrose solution at 4 °C for 48 h. 40 μm-thick coronal sections were cut on a freezing microtome (CM3050 S, Leica) and collected in cold phosphate buffer saline (PBS, 0.01 M, pH 7.4). Slices underwent three more wash steps of 10 min each in PBST, followed by mounting and coverslipping on microscope slides. The extents of virus expression and placements of optrode, and infusion guide cannula were carefully checked, and their images were acquired using an Olympus BX53F fluorescence microscope (Japan) using a 2 × air objective.

### Statistical Analysis

All of the data were expressed as the mean ± standard error of the mean (s.e.m.). The statistical significance was determined by a two-tailed paired Student’s *t*-test, or by a two-way ANOVA with repeated measures followed by Tukey post-hoc test using the SPSS software for the Windows package (v. 18.0). A value of *P* < 0.05 was considered to be statistically significant.

## Supplementary information

Supplementary Information
