# Supplementary material for: Reevaluating the ability of cerebellum in associative motor learning
Source: Sci Rep. 2019 Apr 15;9:6029. doi: 10.1038/s41598-019-42413-5 (PMC6465343; doi:10.1038/s41598-019-42413-5)
Supplement: Supplementary file 1 — Supplementary Information [file 41598_2019_42413_MOESM1_ESM.doc]

**Supplementary Information**

**Reevaluating** **the ability of cerebellum in associative motor learning**

Da-bing Li^1,2,*^, Juan Yao^1,3*^, Lin Sun^1,*^, Bing Wu^1^, Xuan Li^1^, Shu-lei Liu^1^, Jing-ming Hou^4^,

[Hong-liang Liu](https://onlinelibrary.wiley.com/action/doSearch?ContribAuthorStored=Liu%2C+Hongliang)^4^, Jian-feng Sui^1,3^, Guang-yan Wu^1,3^

^1^Experimental Center of Basic Medicine, College of Basic Medical Sciences, Army Medical University, Chongqing 400038, China;

^2^Department of Physiology, School of Basic Medical Sciences, Southwest Medical University, Luzhou Sichuan, 646000, China;

^3^Department of Physiology, College of Basic Medical Sciences, Army Medical University, Chongqing 400038, China;

^4^Department of Rehabilitation, Southwest Hospital, Army Medical University, Chongqing, 646000, China

*These authors contributed equally to this work.

Address correspondence to Guang-Yan Wu, Email: [wgy009@163.com](mailto:wgy009@163.com); Jian-feng Sui, Email: jfsui2003@163.com.

**Supplementary Figure**

**
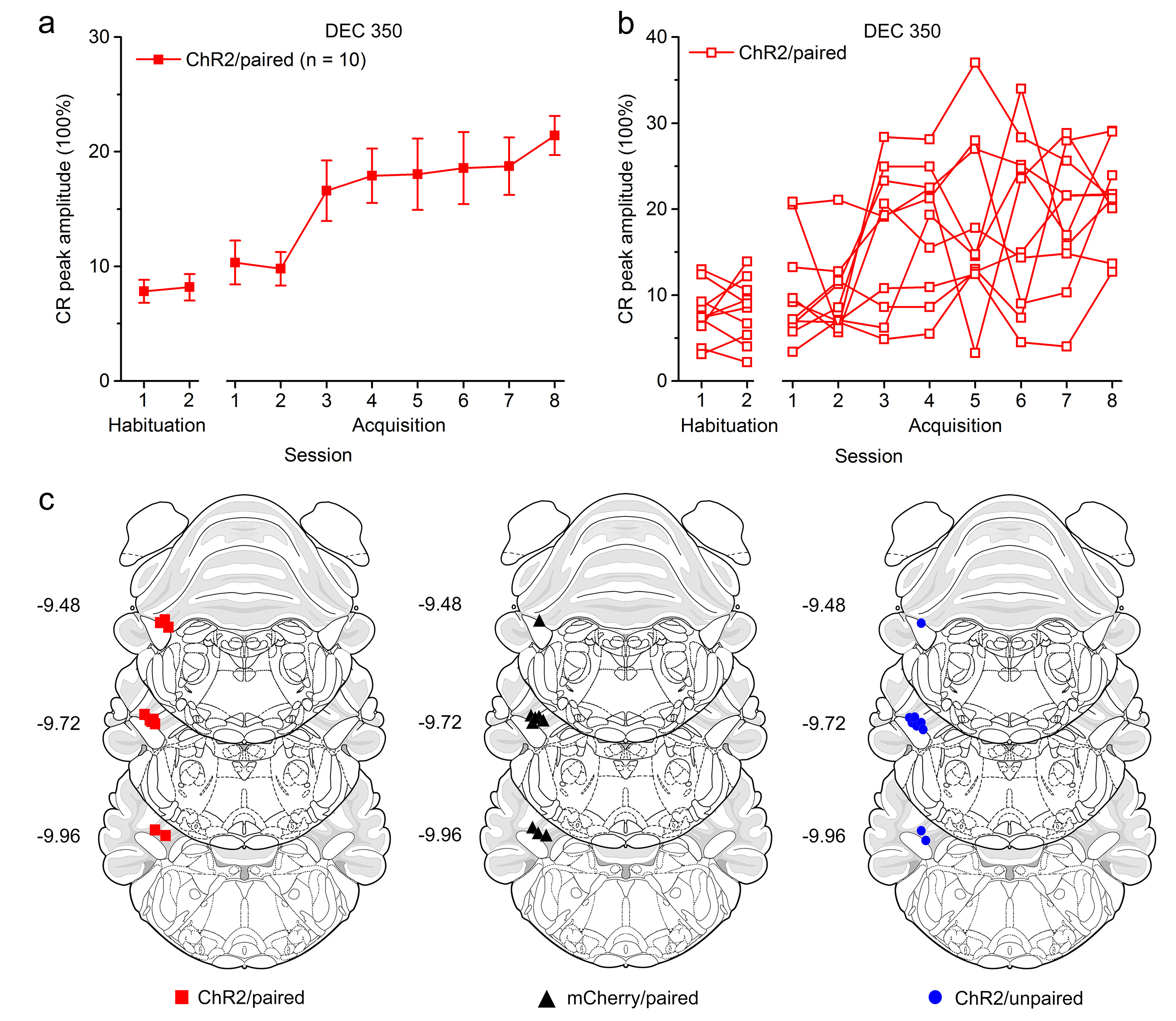
**

**Supplementary Figure S1 | CR peak amplitude for learned rats and placements of optrode in DEC 500 experiment** (related to Fig. 2). (**a**) Average CR peak amplitude of the ChR2/paired (learned) rats. (**b**) CR peak amplitude of ChR2/paired (learned) rats. (**c**) Approximate locations of optrode tips for rats of ChR2/paired, mCherry/paired, and ChR2/unpaired groups. Numbers indicate the anteroposterior coordinates from bregma.


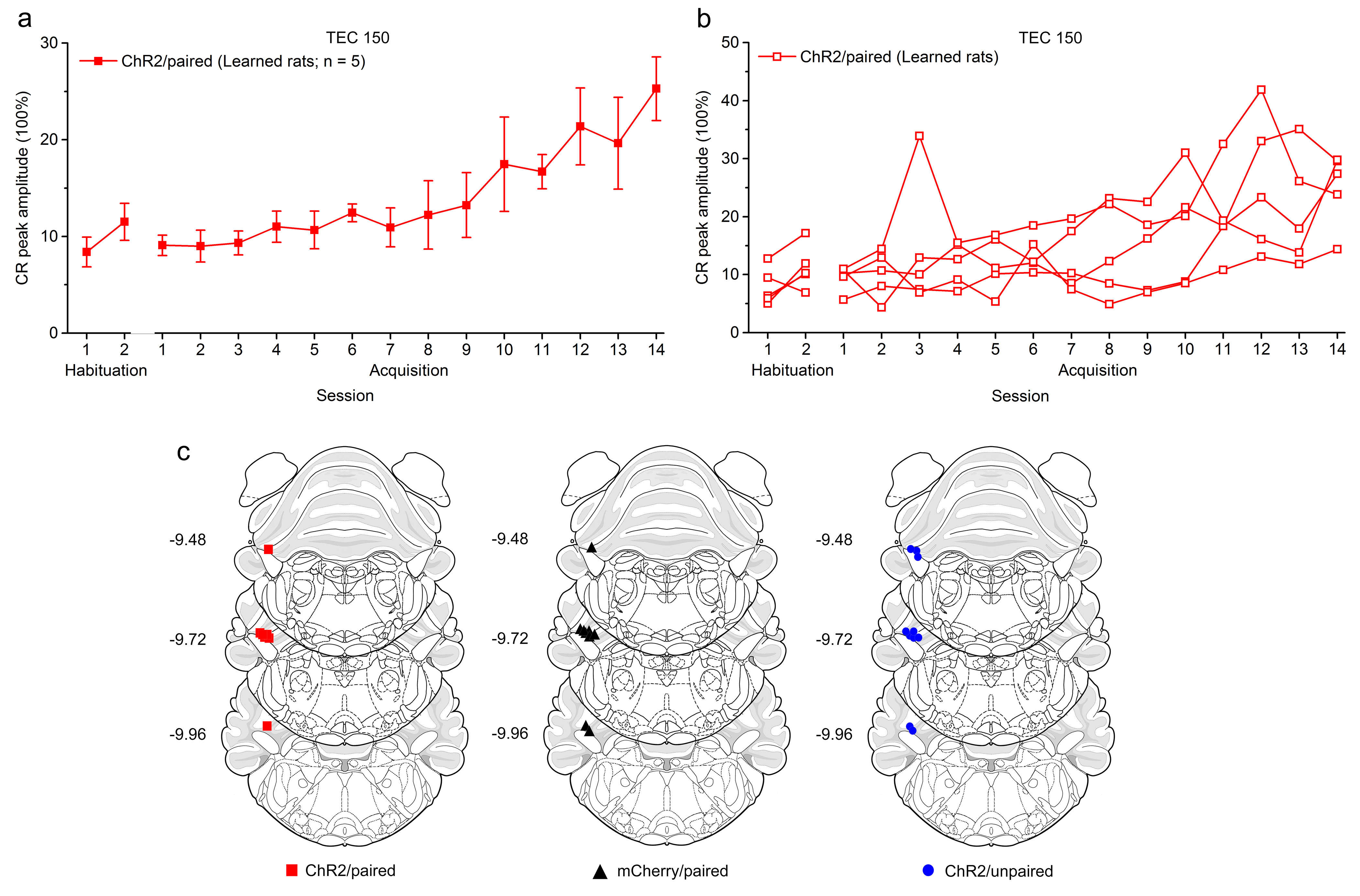


**Supplementary Figure S2 | CR peak amplitude for learned rats and placements of optrode in TEC with a 150-ms trace interval experiment** (related to Fig. 3). (**a**) Average CR peak amplitude of the ChR2/paired (learned) rats. (**b**) CR peak amplitude of ChR2/paired (learned) rats. (**c**) Approximate locations of optrode tips for rats of ChR2/paired, mCherry/paired, and ChR2/unpaired groups. Numbers indicate the anteroposterior coordinates from bregma. Data are represented as mean ± s.e.m.

**
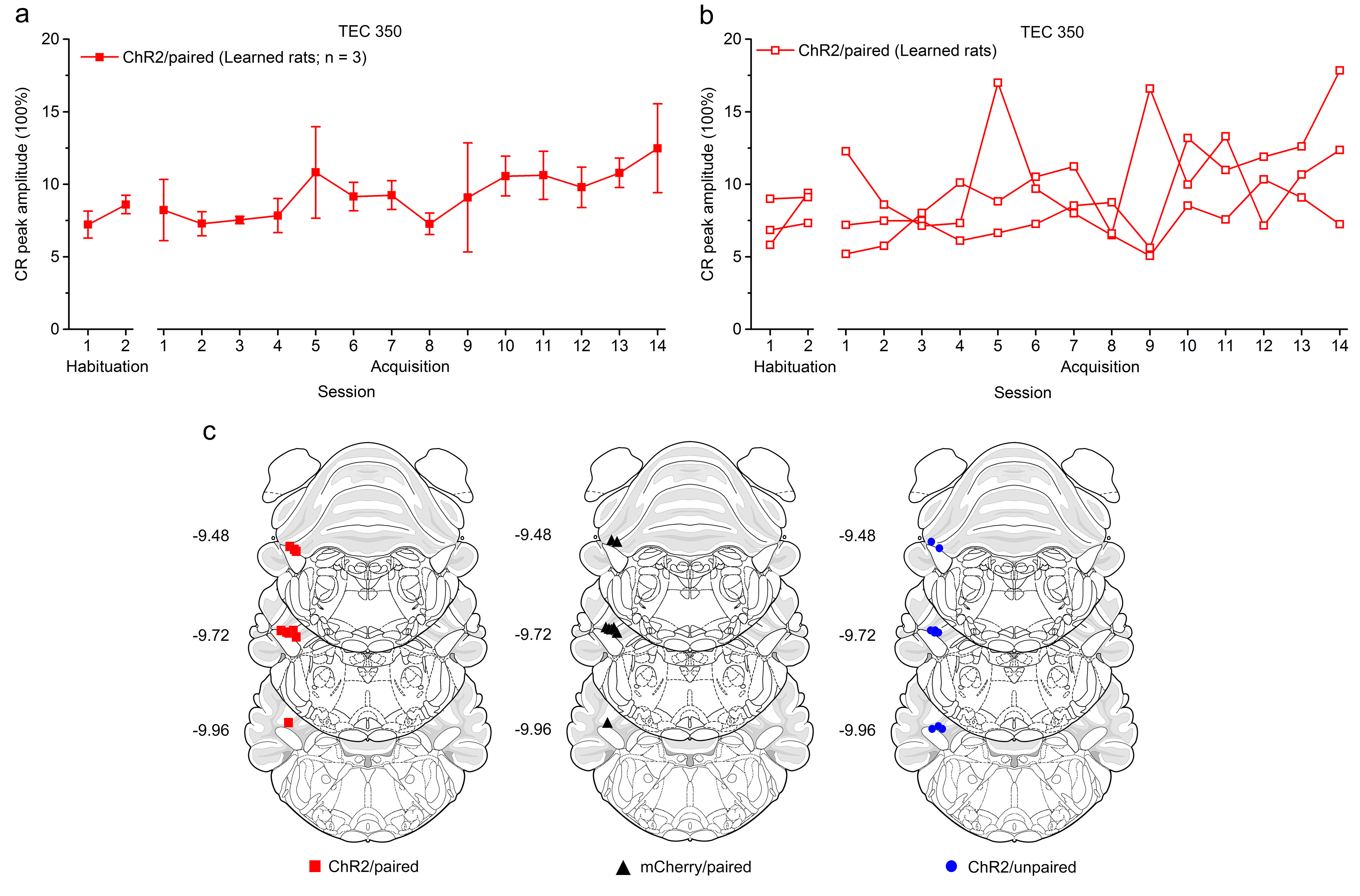
**

**Supplementary Figure S3 | CR peak amplitude for learned rats and placements of optrode in TEC with a 350-ms trace interval experiment** (related to Fig. 4). (**a**) Average CR peak amplitude of the ChR2/paired (learned) rats. (**b**) CR peak amplitude of ChR2/paired (learned) rats. (**c**) Approximate locations of optrode tips for rats of ChR2/paired, mCherry/paired, and ChR2/unpaired groups. Numbers indicate the anteroposterior coordinates from bregma. Data are represented as mean ± s.e.m.

**
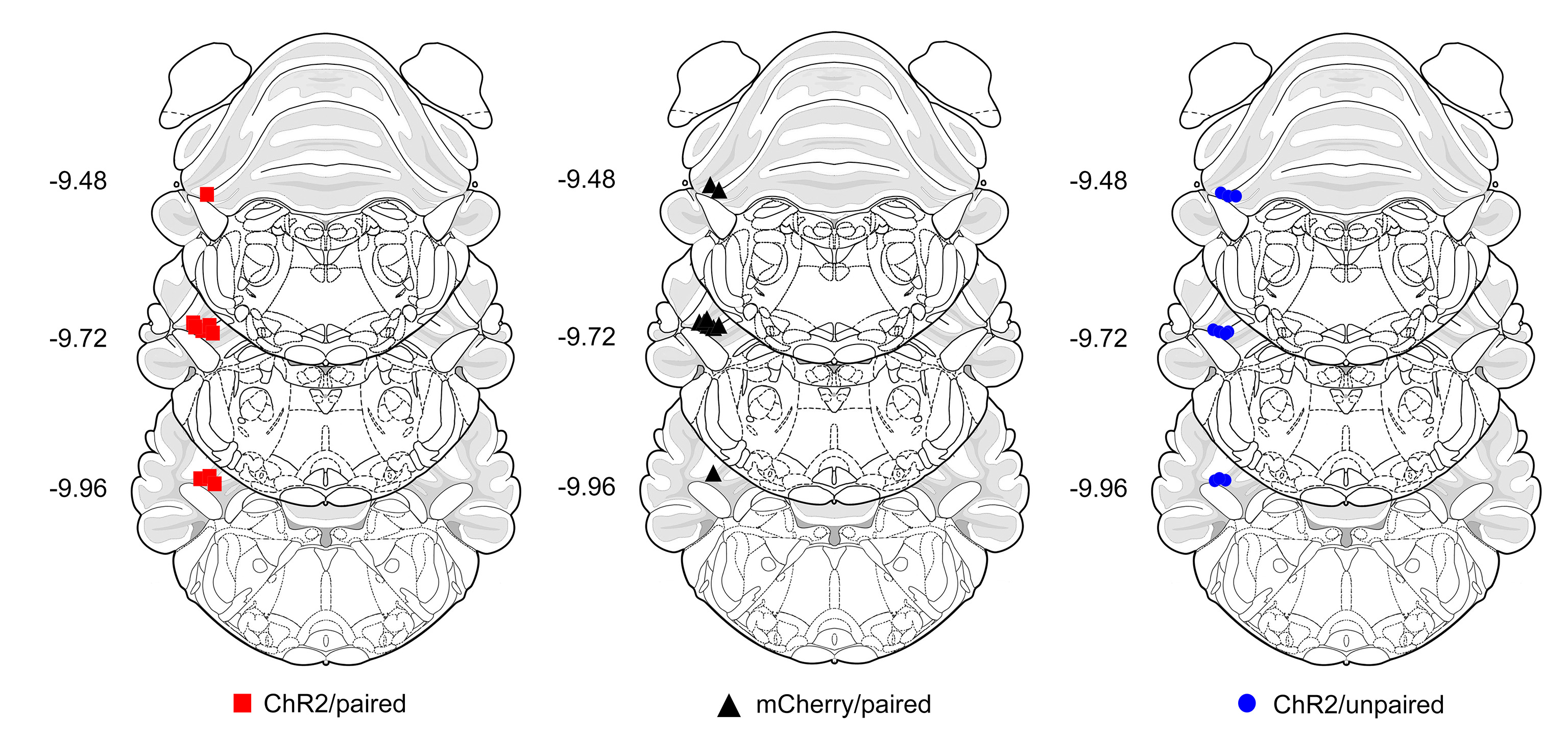
**

**Supplementary Figure S4 | Placements of optrode in TEC with a 500-ms trace interval experiment** (related to Fig. 5). Approximate locations of optrode tips for rats of ChR2/paired, mCherry/paired, and ChR2/unpaired groups. Numbers indicate the anteroposterior coordinates from bregma. Data are represented as mean ± s.e.m.
